# Frequency of limitations statements in original research articles of United States leading medical journals: A meta-research protocol

**DOI:** 10.1371/journal.pone.0305970

**Published:** 2024-11-01

**Authors:** Nin-Chieh Hsu, Hung-Bin Tsai, Chia-Hao Hsu, Ming-Yan Tsai, Charles Liao, Yasuharu Tokuda

**Affiliations:** 1 Division of Hospital Medicine, Department of Internal Medicine, Taipei City Hospital Zhongxing Branch, Taipei, Taiwan; 2 Department of Internal Medicine, College of Medicine, National Taiwan University, Taipei, Taiwan; 3 Department of Orthopedics, Kaohsiung Medical University Hospital, Kaohsiung, Taiwan; 4 College of Medicine, Kaohsiung Medical University, Kaohsiung, Taiwan; 5 Department of Internal Medicine, Stanford University, School of Medicine, Stanford, California, United States of America; 6 Muribushi Okinawa Clinical Training Center, Urasoe City, Okinawa, Japan; 7 Tokyo Foundation for Policy Research, Roppongi, Minato-ku, Tokyo, Japan; Universal Scientific Education and Research Network, UNITED KINGDOM

## Abstract

**Background:**

Limitation declarations are commonly deemed essential to uphold intellectual humility for scientific research, but little has been reported about the limitation statements in published original research articles. This meta-research study aims to investigate the trends of limitation statements among three leading general medical journals in the US.

**Methods:**

This cross-sectional study will compile a data set of full-length original research articles published in the New England Journal of Medicine, Journal of the American Medical Association, and Annals of Internal Medicine between 2002 and 2022. Limitation statement will be recognized by two investigators, and a predefined set of sensitive keywords is used for sensitivity analysis. Frequency of limitation statements within the main text of research articles and trends for different study designs, including their association with the corresponding reporting guidelines, are the main measurements. We employ the Cochran-Armitage test for trend analysis.

**Conclusion:**

The findings of this study will provide an overview of the limitation statements in leading general medical journals in the US. The results may contribute to future research to identify factors that are associated with the presence of limitation statements.

## Introduction

The concept of intellectual humility is gaining considerable recognition, celebrated as an integral aspect of wisdom, a facilitator of self-improvement, and a catalyst for more constructive dialogue [1]. Acknowledgement of limitation is an attitude of humility warranted in the scientific research. Reducing potential confounding through meticulous study design is a task assigned to researchers [2, 3]; however, biases remain unavoidable [4, 5]. In accordance with journal author guidelines, providing a statement of limitations is typically obligatory in original research. Nevertheless, the prevalence and pattern of such limitations declarations in published works have not been reported.

The reporting guidelines for pertinent publication types were established approximately 15 years ago [6, 7]. The Consolidated Standards of Reporting Trials (CONSORT) guidelines for randomized control trials (RCT) mandate limitations, generalizability interpretation in the discussion or comment section [6, 8]. The Strengthening the Reporting of Observational Studies in Epidemiology (STROBE) statement for observational studies mandate key results, limitations, interpretation, generalizability in the discussion section [9]. The Preferred Reporting Items for Systematic Reviews and Meta-Analyses (PRISMA) statement for reporting systematic reviews and meta-analyses of studies, consists of a 27-item checklist. also mandate an item to include discussions of limitations at study, outcome, and review level [10].

Despite the explicit requirement for a limitation statement outlined in the respective checklists of these reporting guidelines, adherence to such guidelines is seldom reported. A review of the first 50 articles from each of the six most-cited research journals and two leading open-access journals published in 2005 found that only 67 articles (17%) of the 400 articles mentioned any limitations in their scientific work [11]. However, most of these reports were completed before the implementation of reporting guidelines. Including journals with different editorial policies may introduce bias. Furthermore, previous studies did not examine any trends in the inclusion of limitation statements in articles.

This study aims to assess the frequency of limitation statements in original articles within three leading general medical journals in the United States. It seeks to explore trends from 2002 to 2022, both before and after the publication of relevant reporting guidelines for the respective publication types. Based on personal observations, we hypothesize that there is an increasing trend in the inclusion of limitation statements, particularly after the publication of reporting guidelines.

## Methods and analysis

So far, there is no formal reporting guideline for meta-analysis. We will use the reporting guideline proposed by Murad et al. [12], which provided a framework for reporting meta-epidemiological studies.

### Eligibility criteria

The eligible criteria are original articles published in the New England Journal of Medicine (NEJM), Journal of American Medical Association (JAMA) and Annals of Internal Medicine (Ann Intern Med). These three journals are selected because they were rated the top three general medical journals in the US.

### Study selection

NEJM, JAMA and Ann Intern Med adapt different kinds of columns, consisting of a variety of article types. Therefore, we only enroll articles which were published in the original article section of NEJM, the original contribution and original research section of JAMA, and original research section of Ann Intern Med. Articles categorized in other sections, such as preliminary communication or special communication, regardless of publication types, are not enrolled. A model of flow diagram that will be used to describe for the study selection process is depicted in **S1 Fig** in the supplement file.

### Data collection process

We systematically acquired, through hand-searching, all the full text of original articles published between the first issue in January 2002 to the last issue in December 2022 from the website of the NEJM, JAMA and Ann Intern Med.

We only exclude original articles, by hand-searching, which have been labelled as retracted, regardless of the reason and availability on the journal website. Examples are Mehra’s and Gander’s articles in NEJM and JAMA, respectively [13, 14].

### Data items

The Japan-US-Taiwan meta-research group gathers researchers who have published a few commentaries in both NEJM [15], JAMA [16] and other leading journals [17]. In this study, two reviewers (Hsu NC and Hsu CH) will conduct the investigations independently for each article to determine whether a limitation statement exists. Discrepancies will be resolved by consultation with the corresponding author (Tokuda Y). We identify limitations exclusively within the discussion section, as we observe that articles often discussed methods for minimizing bias or controlling for confounding in the methodology section. Some authors may subjectively characterize these approaches as strengths of the study, while others acknowledge them as limitations. Consequently, our focus is specifically on determining whether these statements are presented in the discussion section.

Variables in this study include the study design and the temporal relationship with the respective reporting guidelines. The study designs are classified into three categories. A randomized controlled trial (RCT) is defined as a comparative study reporting a random assignment of participants anywhere in the report. A clinical trial without random assignment is identified as a non-randomized trial. A meta-analysis study is defined as a quantitative research synthesis method that statistically combines the results of multiple independent studies, typically indicated in the title of a report [18].

The CONSORT statement was first published in 1996 [6], with subsequent revision in 2001 [7], and 2010 [8]. We investigate publication between 2002 and 2022 and chose the year 2010 as a reference point to assess the pre-post changes in limitation statements of randomized trials. The STROBE guideline for observational studies, initially published in 2007 without subsequent revisions [10], serves as the reference point for pre-post changes in limitation statements for non-randomized trials.

### Measures

The main measure of our study is the existence of limitation statements in eligible articles. Before investigation, the authors have convened a consensus meeting regarding the definition of the limitation statement. The forms of the limitation statement are categorized and exemplified in **S1 Table**. After a random review of original articles published in these three journals, there are ten primary forms of limitation statements commonly found in the discussion section of academic papers, which include: (1) a subheading entitled “limitations”; (2) a subheading entitled “strengths and limitations”; (3) a subheading entitled “methodological considerations”; (4) a single paragraph discussing limitations without a subheading; (5) multiple paragraphs discussing limitations without a subheading; (6) statements of limitations or weaknesses alongside strengths within the same paragraph; (7) discussions of “bias” without mentioning limitations; (8) discussions of “weakness” without mentioning limitations; (9) reasons for interpreting the results cautiously, without directly mentioning limitations; and (10) acknowledgments of areas where the study may fall short, without using the terms “limitations,” “weakness,” or “bias.” (**S1 Table**)

### Synthesis of results

The main result of interest is the frequency of limitation statement in the discussion section of eligible original research articles, and the trend of annual publications from 2002 to 2022 counted on a yearly basis.

To avoid missing limitation statements in articles not reporting limitations in any of the ten forms mentioned above, we will conduct a sensitivity analysis. Three reviewers (Hsu NC, Hsu CH, and Tsai HB) have convened a consensus meeting to identify additional words that will be used during the sensitivity analysis: "limited", "confound", and "caution". Combined with “limitation”, “weakness”, and “bias”, which we have already employed in the primary form, the sensitivity analysis will contain six words. We have done a pilot testing of the sensitivity analysis using the original articles published in the first three months of 2012, 2014, 2016, 2018, 2020 and 2022 in NEJM and JAMA. With the gold standard of limitation statements recognized independently by two reviewers (Hsu NC and Hsu CH), the absence of all six limitation-pertinent words predicted no limitation statement with 100% accuracy. The occurrence of at least two of the six words resulted in a 100% accuracy in predicting the existence of limitation statements. Based on this pilot test, articles lacking any of the six limitation-pertinent words will be categorized as having no limitation statements in this study. On the websites of these journals, we will use these limitation-pertinent words to manually screen the full text of each article in electronic form as a sensitivity analysis.

We use the Chi-square test to depict frequency distributions for descriptive categorical variables, such as the frequency of limitation statements within a specific journal or a research design. Trends between journals, research designs, and pre-post change are analyzed by the Cochran Armitage test with a P value. Analyses will be conducted using R (R Project for Statistical Computing version 4.1.2) and SPSS version 24. A P value of 0.05 or less will be used to define the statistical significance of a test result.

We do not seek approval from the research ethics committee or institutional review board because the study does not involve human subjects, and the data are publicly available.

## Discussion

The fourth edition of the "Uniform Requirements for Manuscripts Submitted to Biomedical Journals," initially published in NEJM in 1991, served as a coordinating document developed by the International Committee of Medical Journal Editors (ICMJE) in North America [19]. In this document, it suggested the article should “include in the Discussion section the implications of the findings and their limitations, including implications for future research”. Because the limitations of a study can be discussed in various ways, it becomes challenging to audit their presence or absence.

From the introduction of the first CONSORT statement in 1996, statements of specific interpretation of study findings, including sources of bias and imprecision (internal validity) and discussion of external validity, including appropriate quantitative measures are necessary in the comment section among the five structured subheadings of RCT [6]. However, the submission process of an original research for some leading medical journals does not mandate an inclusion of any formal reporting guideline.

Our study aims to analyze the inclusion of limitation statements in the reports of original research published by three leading general medical journals in the United States. We hypothesize that the inclusion of limitation statements has been increasing, particularly following the publication of reporting guidelines. This investigation may contribute to the audit of reporting quality in trials, compatible with the goal and mission of a meta-research study. A previous review in psychology critically examined diverse approaches to defining and measuring intellectual humility and identified the common element: a meta-cognitive ability to recognize the limitations of one’s beliefs and knowledge [1]. Therefore, limitation statements in a research report are crucial and go beyond merely adhering to reporting guidelines.

### Limitations

Potential limitations of our study encompass exclusive focus on three medical journals in the US, challenges in recognizing limitation statements, variations in author writing styles, confounding by the peer review process, and absence of a comprehensive analysis of the content and quality of the limitation statements. NEJM doesn’t require the inclusion of a subsection on limitations in the discussion. While JAMA suggested a subsection of limitation since 2017, we emphasize that our study refrains from drawing direct comparisons between the journals in this context.

## Conclusion

The findings of this study may provide an overview of the limitation statements in leading general medical journals in the US, including the frequency and trends. As this is an inaugural investigation, the results may provide a foundation for future research aimed at identifying factors such as article types, research topics, and journal author guidelines that are associated with the presence of limitation statements.

## Supporting information

S1 Fig A modified PRISMA flow diagram for meta-research analysis. (DOCX)

S1 Table Forms of limitation statement in the discussion section of original research articles. (DOCX)

## References

[1] Porter T, Elnakouri A, Meyers EA, Shibayama T, Jayawickreme E, Grossmann I. Predictors and consequences of intellectual humility. Nat Rev Psychol. 2022;1(9):524–536. doi: 10.1038/s44159-022-00081-9 35789951 PMC9244574

[2] Mamdani M, Sykora K, Li P, et al. Reader’s guide to critical appraisal of cohort studies: 2. Assessing potential for confounding. BMJ. 2005;330(7497):960–962. doi: 10.1136/bmj.330.7497.960 15845982 PMC556348

[3] Kahan BC, Morris TP. Reporting and analysis of trials using stratified randomisation in leading medical journals: review and reanalysis. BMJ. 2012;345:e5840. Published 2012 Sep 14. doi: 10.1136/bmj.e5840 22983531 PMC3444136

[4] Yadav K, Lewis RJ. Immortal Time Bias in Observational Studies. JAMA. 2021;325(7):686–687. doi: 10.1001/jama.2020.9151 33591334

[5] Holmberg MJ, Andersen LW. Collider Bias. JAMA. 2022;327(13):1282–1283. doi: 10.1001/jama.2022.1820 35285854

[6] Begg C, Cho M, Eastwood S, et al. Improving the Quality of Reporting of Randomized Controlled Trials: The CONSORT Statement. JAMA. 1996;276(8):637–639. doi: 10.1001/jama.1996.03540080059030 8773637

[7] Hopewell S, Dutton S, Yu LM, Chan AW, Altman DG. The quality of reports of randomised trials in 2000 and 2006: comparative study of articles indexed in PubMed. BMJ. 2010;340:c723. doi: 10.1136/bmj.c723 20332510 PMC2844941

[8] Schulz KF, Altman DG, Moher D; CONSORT Group. CONSORT 2010 Statement: updated guidelines for reporting parallel group randomised trials. BMC Med. 2010;8:18. Published 2010 Mar 24. doi: 10.1186/1741-7015-8-18 20334633 PMC2860339

[9] von Elm E, Altman DG, Egger M, et al. The Strengthening the Reporting of Observational Studies in Epidemiology (STROBE) statement: guidelines for reporting observational studies. Lancet. 2007;370(9596):1453–1457. doi: 10.1016/S0140-6736(07)61602-X 18064739

[10] Liberati A, Altman DG, Tetzlaff J, et al. The PRISMA statement for reporting systematic reviews and meta-analyses of studies that evaluate healthcare interventions: explanation and elaboration. BMJ 2021;372:n71 doi: 10.1136/bmj.n71 19622552 PMC2714672

[11] Ioannidis JP. Limitations are not properly acknowledged in the scientific literature. J Clin Epidemiol. 2007;60(4):324–329. doi: 10.1016/j.jclinepi.2006.09.011 17346604

[12] Murad MH, Wang Z. Guidelines for reporting meta-epidemiological methodology research. Evid Based Med. 2017;22:139–142. doi: 10.1136/ebmed-2017-110713 28701372 PMC5537553

[13] Mehra MR, Desai SS, Kuy S, Henry TD, Patel AN. Cardiovascular Disease, Drug Therapy, and Mortality in Covid-19 [retracted in: N Engl J Med. 2020 Jun 4;:]. N Engl J Med. 2020;382(25):e102. doi: 10.1056/NEJMoa2007621 32356626 PMC7206931

[14] Gander JC, Zhang X, Ross K, et al. Association Between Dialysis Facility Ownership and Access to Kidney Transplantation. JAMA. 2019;322(10):957–973. doi: 10.1001/jama.2019.12803 31503308 PMC6737748

[15] Hsu NC, Hsu CH. Anticoagulation with Edoxaban in Patients with Atrial High-Rate Episodes. N Engl J Med. 2023;389(24):2301–2302. doi: 10.1056/NEJMc2312837 38091538

[16] Hsu NC, Yang HL, Hsu CH. Challenges to Door-In-Door-Out Time Thresholds for Patients With Stroke. JAMA. 2023;330(23):2305–2306. doi: 10.1001/jama.2023.21488 38112819

[17] Hsu NC, Liao C, Hsu CH. Proportions and trends of critical care trials in leading general medical journals, 1970–2022. Crit Care. 2023;27(1):375. doi: 10.1186/s13054-023-04666-5 37773187 PMC10540386

[18] McInnes MDF, Moher D, Thombs BD, et al. Preferred Reporting Items for a Systematic Review and Meta-analysis of Diagnostic Test Accuracy Studies: The PRISMA-DTA Statement. JAMA. 2018;319(4):388–396. doi: 10.1001/jama.2017.19163 29362800

[19] International Committee of Medical Journal Editors. Uniform requirements for manuscripts submitted to biomedical journals. N Engl J Med. 1991;324(6):424–428. doi: 10.1056/NEJM199102073240624 1987468

