# Supplementary figures and images for: Frequency of limitations statements in original research articles of United States leading medical journals: A meta-research protocol
Source: PLoS One. 2024 Nov 1;19(11):e0305970. doi: 10.1371/journal.pone.0305970 (PMC11530002; doi:10.1371/journal.pone.0305970)

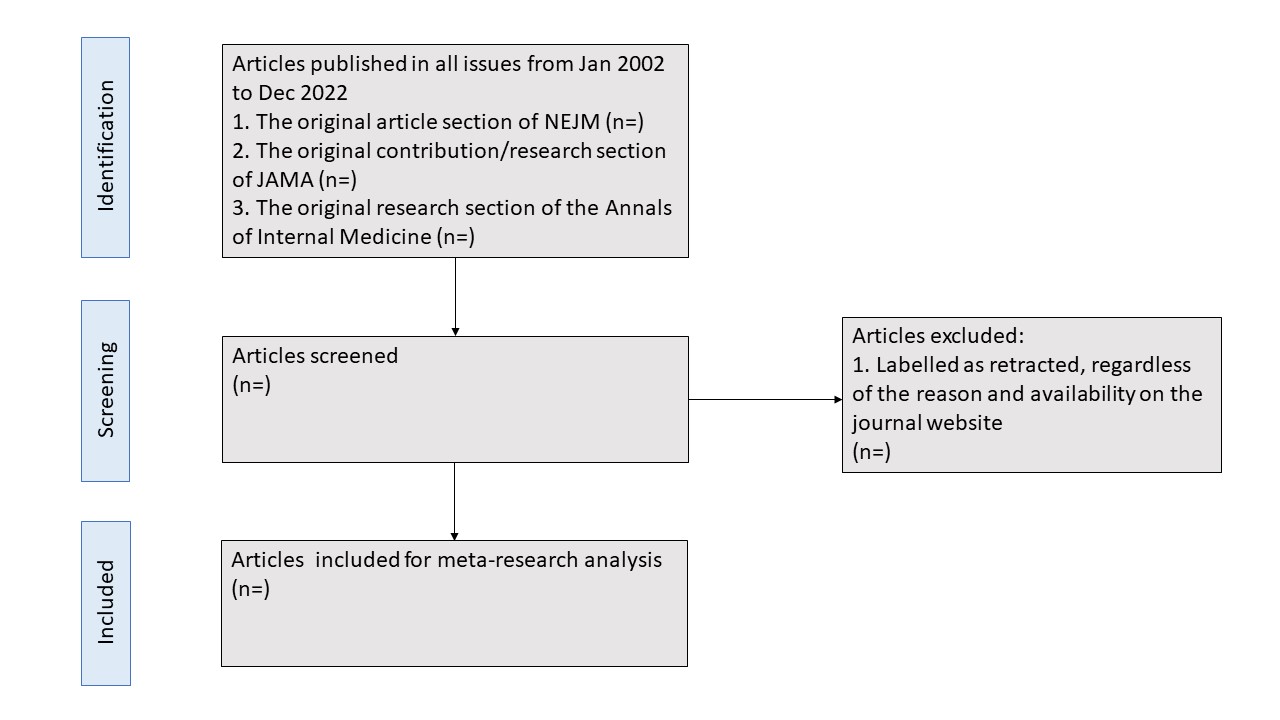


**Supporting eFigure 1**. A modified PRISMA flow diagram for meta-research analysis.

Supplement: S1 Fig — (DOCX) [file pone.0305970.s001.docx]
