# Supplementary material for: Frequency of limitations statements in original research articles of United States leading medical journals: A meta-research protocol
Source: PLoS One. 2024 Nov 1;19(11):e0305970. doi: 10.1371/journal.pone.0305970 (PMC11530002; doi:10.1371/journal.pone.0305970)
Supplement: S1 Table — (DOCX) [file pone.0305970.s002.docx]

**Supporting eTable 1**. Forms of limitation statement in the discussion section of original research articles

| **Forms** | **Examples and references** |
| --- | --- |
| With a subheading entitled “limitations” | Collins S, et al. JAMA. 2014;311(11):1125-1132.  Plevritis SK, et al. JAMA. 2018;319(2):154-164. |
| With a subheading entitled “strengths and limitations” | Bieleninik L, et al. JAMA. 2017;318(6):525–535. |
| With a subheading entitled “methodological considerations” | Besseling J, et al. JAMA. 2015;313(10):1029–1036. |
| A single paragraph discussing limitations without a subheading | Cho BH, et al. Ann Intern Med. 2022;175(1):1-10.  Feldman TE, et al. JAMA. 2018;319(1):27-37. |
| More than one paragraph discussing limitations without a subheading | Herweijer E, et al. JAMA. 2014;311(6):597-603.  Eyre DW et al. N Engl J Med 2022; 386:744-756 |
| Limitation or weakness statements alongside strengths in the same paragraph | Dawwas GK, et al. Ann Intern Med. 2022;175(1):20-28.  Behrens I, et al. JAMA. 2016;315(10):1026–1033 |
| Discuss “bias” without mentioning limitations | Sørup S, et al. JAMA. 2014;311(8):826–835 |
| Discuss “weakness” without mentioning limitations | Weintraub ES, et al. N Engl J Med 2014; 370:513-519 |
| State reasons that the results should be interpreted cautiously, without directly mentioning limitations | Fijen LM, et al. N Engl J Med 2022; 386:1026-1033  Gheorghiade M, et al. JAMA. 2013;309(11):1125–1135. |
| Acknowledge the areas where the study may fall short, without using the terms "limitations," "weakness," or "bias." | Houssami N, et al. JAMA. 2011;305(8):790–799. (“should be interpreted with consideration of possible confounding”)  Li BT, et al. N Engl J Med 2022; 386:241-251. (lack of a comparator group) |
